# Intra-rater and Inter-rater Reliability of the Commander Pressure Algometer in Greek Patients With Chronic Neck Pain

**DOI:** 10.7759/cureus.66350

**Published:** 2024-08-07

**Authors:** Charalampos Skordis, Christina Liaskou, Evangelia Papagiakoumou, Spyridon Sotiropoulos, Theodora Plavoukou, Palina Karakasidou, George Georgoudis

**Affiliations:** 1 Physiotherapy Department, University of West Attica (UNIWA), Athens, GRC

**Keywords:** commander, non-specific chronic neck pain, greek patients, inter-rater reliability, intra-rater reliability, pressure algometer, pressure pain threshold (ppt)

## Abstract

Introduction

Non-specific chronic neck pain (NSCNP) is a musculoskeletal disorder that affects 45%-54% of the general population. There is a strong correlation between patient-reported pain and mechanical pain pressure threshold (PPT) measured with an algometer.

Purpose

This study aims to investigate the intra- and inter-rater reliability of the Commander algometer in Greek NSCNP patients, in an urban primary care setting.

Methods

Thirty-three patients (22 women and 11 men) suffering from NSCNP (>3 months), the majority (42.4%) between the ages of 50 years and 59 years and overweight, were measured bilaterally both at the neck (mastoid, trapezius head-insertion and mid-portion, C5-C6 facet, insertion of levator scapula) and at the control areas (mid-deltoid and tibialis anterior) using the Commander^ ^algometer. Measurements were taken twice over a span of six days, by two raters, in a primary care setting. Intraclass correlation coefficient (ICC) statistics were used as measures of reliability (p = 0.05).

Results

Intra-rater reliability was “moderate to good” for both raters. ICC values for PPT at the seven bilaterally measured sites varied between 0.67 and 0.86 for the first rater (p ≤ 0.001) and 0.64 and 0.82 for the second rater (p ≤ 0.003). The inter-rater reliability was “moderate to excellent” (ICC = 0.68-0.92) in the first measurement (T1) and “moderate to good” (ICC = 0.68 to 0.89) in the second measurement (T2).

Conclusion

This study supports the intra- and inter-rater reliability of the Commander algometer in detecting reliably the mechanical PPT, in Greek NSCNP patients, as measured according to the procedures and methodology followed throughout this study.

## Introduction

Non-specific chronic neck pain (NSCNP) or mechanical neck pain are the most common terms used to define pain in the lateral and posterior neck [1]. It is a musculoskeletal disorder affecting 45%-54% of the general population at least once in their lives [2]. A strong correlation between the reported pain and the level of measured mechanical sensitivity has been reported [3,4]. Hyperalgesia can occasionally be found in anatomical regions distant from the local site of injury [3], which is an indicator of central sensitization [5]. According to a systematic review and meta-analysis, 79% of the included studies selected the tibialis anterior muscle as a remote algometric site, assessing central pain sensitization [5].

A pressure algometer is an instrument used to measure sensitivity to pain through the application of pressure [6]. The pressure algometer’s ability to measure sensitivity can contribute to the evaluation of treatment results, the recognition of myofascial trigger points (MTrPs), and the quantification of the mechanical pain pressure threshold (PPT) [7].

The Commander pressure algometer is a fairly new but popular clinical practice device. However, a limited number of accessible studies were found to use this algometer for research [8-11], out of which, only one examined the reliability of PPT measurements in 100 healthy young adults. It was applied on the supraspinatus tendon, the anterior talofibular ligament, and the extensor digitorum communis muscle belly of the dominant side. High intra-rater reliability (Cronbach’s alpha values range > 0.85) was found on these sites. On the contrary, the inter-rater reliability was poor to moderate (ICC < 0.561) [9].

No published research results could be found in the literature regarding the reliability of the Commander algometer in NSCNP patients. Therefore, the aim of this study was to investigate the intra- and inter-rater reliability of the Commander algometer in Greek patients with chronic neck pain.

## Materials and methods

Study design

This study was a single-group reliability study with repeated measurements. For further investigation of the reproducibility and validity of the algometer measurements, two independent raters measured the mechanical sensitivity in a group of NSCNP patients. Measurements were carried out in two instances over a span of six days.

This study was performed at the “Mikis Theodorakis” Multipurpose Center for Cultural, Sports and Social Activities of Ilion in Athens, Greece, in collaboration with the Musculoskeletal Physiotherapy Research Lab of the University of West Attica (UNIWA) in Athens from January 2023 to August 2023. All measurements were taken by two independent and experienced pressure-algometry physiotherapists. The study has been submitted and approved by the Research Ethics Committee of the University of West Attica (UNIWA), with protocol number 103276/18-12-2020.

Participants

Thirty-three patients (N = 22 women) suffering from NSCNP (≥3 months), aged 18-70, were included. The exclusion criteria were neck pain related to neurological disorders, systematic inflammatory disease or rheumatic diseases, or another known pathological cause, previous surgery, or any kind of trauma at least two years ago. Patients receiving other treatments during the study were also excluded.

Procedure

Prior to algometry measurements, four questionnaires were administered to the patients. Because of their neck pain and symptom reported variability [12,13], these questionnaires aimed at assessing their perceived change in disability and pain at different time points [14], depressive and anxiety symptoms [15], their multidimensional aspects of pain [16], and their kinesiophobia [17]. Demographic data and usage of other medications were also recorded.

The measurements were carried out in the morning, from 09:30 to 12:30 [7], in a stable temperature environment (25°C) and the same office. These factors were maintained as constant as possible during the measurements [4].

**Figure 1:**
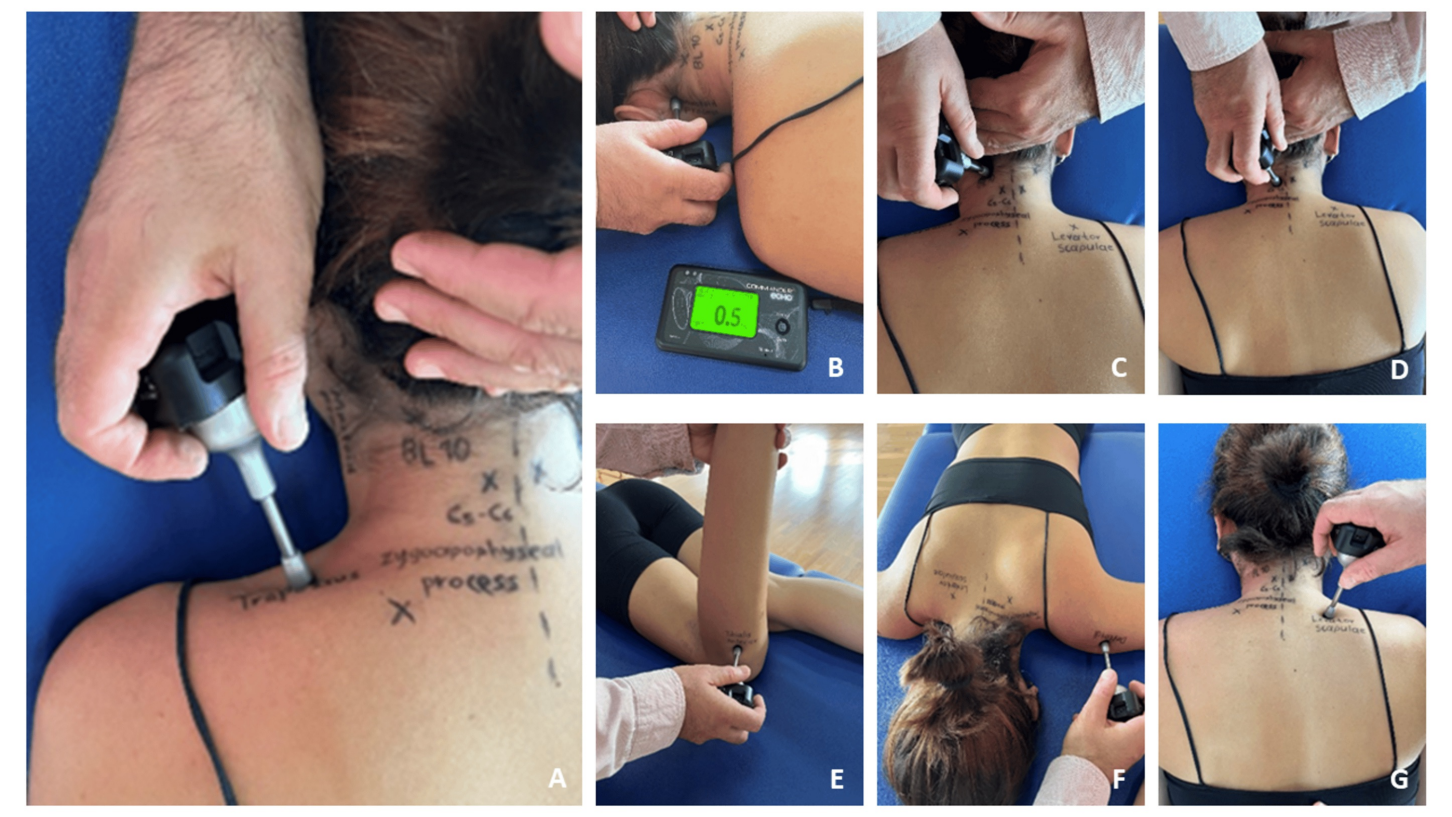
Representation of the measured algometric sites The sites were A) upper trapezius, B) mastoid process, C) bladder 10 (BL 10), D) C5-C6 zygapophyseal joint, E) tibialis anterior, F) deltoid, G) levator scapula.

Several specific sites were selected for the measurements since these were the most popular points of interest in NSCNP. The measurement sites selected were the upper trapezius at the point between the midline and the lateral border of the acromion [18], the suboccipital muscles at the mastoid process, and the bladder 10 (BL 10) acupoint located at the end of the posterior neck hairline and approximately 5 cm lateral to the midline of trapezius muscle [19,20], the zygapophyseal joint between C5-C6 intervertebral space, the tibialis anterior muscle (ST 36) acupoint [4,21], as a remote site (as the first control area), indicator of central sensitization [5], the middle part of the deltoid muscle (1-2 cm below the acromion), as the second control area and the levator scapula muscle (2 cm above its epiphysis, at the upper medial corner of the scapula) [18] (Figure 1).

Participants were placed in the prone position [7] for all measurements. The first measurement was discarded since it was considered a trial [22]. The average of the two consecutive measurements (second and third) was then calculated and recorded as the final value.

The tip of the algometer was perpendicularly applied to the body surface and the rate of pressure was constantly kept at 1 kg/cm^2^ per second, using the visual feedback of the equipment [23,24].

Standardized procedures were followed at all times during the measurements. Specifically, one of the raters, randomly selected, marked all the measurement sites according to the preset list of points and the other measured the patient with the algometer for the first time. After about 20 minutes, which has already been demonstrated to be adequate [22], the second rater performed the same measurements choosing randomly from the measuring points. The whole procedure was repeated after six days [18,25], at the same setting, with the same raters for the same measuring sites but in random order. The expressions the examiners used to inform the patients were standardized without further explanations. The procedures were completed within the same timeframe for each patient and the raters had no access to the data of the patients. The data were transferred to data spreadsheets blindly, and an independent and blind-to-the-procedure statistician did the analyses.

Instruments

Pressure Algometer

The pressure algometer (Commander^®^ algometer, JTECH Medical, Midvale, Utah) was used in all measurements. This particular model is a handheld algometer with two different surface heads (0.5 cm^2^, 1 cm^2^), a flat surface (Flat Pad), and a fingertip adapter. The maximum input force reaches 111 N, while the wireless radio frequency (RF) reaches 2.4 GHz. The surface head used was that of one square centimeter (1 cm^2^) and the unit of measurement for the threshold value was selected to be the kilogram per square centimeter (kg/cm^2^).

Global Perceived Effect

The global perceived effect (GPE) scale rates the patients' perceived change in different domains such as pain and disability. It asks the patient to rate how much their condition has worsened or improved compared to another predetermined point in time [14]. It is a numerical scale that consists of only one question with five possible answers. The GPE scale has shown excellent test-retest reliability with ICC values of 0.90-0.99 [26].

TAMPA Scale Kinesiophobia

The TAMPA scale kinesiophobia (TSK) assesses the fear associated with movement (kinesiophobia) in patients with musculoskeletal pain. The original scale consists of 17 questions [27], each of which is rated on a scale of 1 to 4 points (1 equals “strongly disagree” and 4 equals “strongly agree”). Thus, the final score can range from 17 to 68 points, where 17 corresponds to "no kinesiophobia", whereas 68 to "severe kinesiophobia" [28]. The Greek version was selected for this study since it has shown adequate validity and reliability [29,30].

Hospital Anxiety and Depression Scale

The Hospital Anxiety and Depression Scale (HADS) was created to assess symptoms of depression and anxiety in patients [31]. The HADS scale consists of 14 questions, 7 assess anxiety symptoms and 7 assess depression symptoms, each of which is rated on a scale of 0 to 3 points. The Greek version of the HADS scale has been proven to have high test-retest reliability (ICC = 0.944) and high validity [32].

Short-Form McGill Pain Questionnaire

The short-form McGill pain questionnaire (SFMPQ) expresses perceived pain in the sensory and affective dimensions [33]. It is comprised of 15 descriptive adjectives of the pain sensation 11 of which concern sensory and 4 affective aspects. The patient rates each description on a four-point Likert-type intensity scale ranging from 0 to 3: 0 equals none and 3 equals severe pain [34,35]. The visual analog scale (VAS) and the present pain intensity (PPI) scale are included in the SFMPQ. The VAS scale is a self-report pain measurement scale, where the intensity of the present pain is rated on a scale of 0 to 10. PPI is a six-point rating scale, according to which the patient selects the answer that best describes the pain sensation perceived at the moment, on a scale of 0 to 5 [35]. The total pain score is calculated by the sum of all the intensity values. The Greek version of the short-form McGill pain questionnaire (GR-SFMPQ) whose validity, reliability, and sensitivity are demonstrated, is used throughout this study [35].

Statistical analysis

Quantitative variables were expressed as mean and standard deviation (SD). Qualitative variables were expressed as absolute and relative frequencies. Intraclass correlation coefficient (ICC) values and their 95% confidence intervals were used between raters and between time points (T1/T2). The ICC is a value between 0 and 1, where values below 0.50 indicate poor reliability, between 0.50 and 0.75 moderate reliability, between 0.75 and 0.90 good reliability, while any value above 0.90 indicates excellent reliability [36]. Power analysis was conducted for the determination of the sample size and it was found that to detect an ICC > 0.90 with 80% power, a sample of 33 participants is needed. Scores in SFMPQ, VAS, PPI, HADS, GPE, and TSK scales were compared between T1 and T2 using the Wilcoxon signed-rank test, and levels of kinesiophobia were compared between T1 and T2 using the McNemar test. Moreover, the standard error of measurement (SEM) and the minimal detectable change (MDC) were computed as a measure of absolute agreement expressed in real units of measurement and as the smallest change that can be interpreted as a real difference respectively. All reported p values are two-tailed. Statistical significance was set at p < 0.05 and analyses were conducted using IBM SPSS Statistics for Windows, Version 26 (Released 2019; IBM Corp., Armonk, New York, United States).

## Results

The sample consisted of 33 patients (66.7% women), whose characteristics are presented in Table 1. Most patients (42.4%) were between 50 and 59 years old and overweight. University alumni made up 30.3% of the sample, while 39.4% had completed secondary educational levels. Employees in the public sector were 30.3% of the sample and 69.7% were married. Among the participants 84.8% had pain symptoms for more than two years, 63.4% were under medication and 69.7% suffered from another disease.

**Table 1:** Sample characteristics

		N (%)
Sex		
	Men	11 (33.3)
	Women	22 (66.7)
Age (years)		
	18-29	1 (3)
	30-39	2 (6.1)
	40-49	5 (15.2)
	50-59	14 (42.4)
	60-69	11 (33.3)
BMI (kg/ m^2^), mean (SD)		27.1 (4.5)
BMI categories		
	Normal	11 (33.3)
	Overweight	14 (42.4)
	Obese	8 (24.2)
Educational level		
	Primary	1 (3)
	Secondary	13 (39.4)
	Two-year college	6 (18.2)
	University	10 (30.3)
	MSc/PhD holder	3 (9.1)
Work status		
	Unemployed	7 (21.2)
	Employee in the public sector	10 (30.3)
	Freelancer	1 (3)
	Employee in the private sector	6 (18.2)
	Pensioner	9 (27.3)
Family status		
	Unmarried	8 (24.2)
	Divorced	2 (6.1)
	Married	23 (69.7)
Symptom duration (months)		
	03-Jun	1 (3)
	06-Dec	2 (6.1)
	Dec-24	2 (6.1)
	>24	28 (84.8)
Medication		21 (63.4)
Other disease		23 (69.7)

Participants' scores on the SFMPQ scales and their depression scores were significantly greater at T2 (Table 2). On the contrary, participants’ anxiety score and their scores in VAS, PPI, GPE, and TSK scales were similar in T1 and T2. High levels of kinesiophobia (i.e., TSK score ≥ 37) expressed 36.4% (N = 12) of the sample at T1 and 39.4% (N = 13) at T2; p = 1.000.

**Table 2:** Participants’ scores in McGill, VAS, PPI, HADS, GPE, and TSK scales at T1 and T2 ^*^ p ≤ 0.05; ^**^ p ≤ 0.01 VAS: visual analog scale; PPI: present pain intensity; HADS: Hospital Anxiety and Depression Scale; GPE: global perceived effect; TSK: TAMPA scale kinesiophobia

	T1		T2		
	Mean	SD	Mean	SD	P Wilcoxon sign test
Sensory score	10.36	5.39	13.73	6.45	0.015^*^
Affective score	3.67	3.06	5.15	2.99	0.007^**^
Total McGill score	14.03	7.86	18.88	8.71	0.006^**^
Depression scale (HADS)	9.06	1.73	9.94	1.92	0.025^*^
Anxiety scale (HADS)	10.94	2.34	10.94	1.84	0.848
VAS score	5.09	1.72	5.00	2.11	0.817
PPI score	1.85	0.80	1.94	0.75	0.592
GPE score	3.09	0.52	2.97	0.73	0.405
TSK score	36.00	7.10	35.91	7.26	0.939

ICC values for the between measurements agreement (intra-rater) are presented in Table 3, for each rater separately. More analytically, in both raters, there were significant values detected between T1 and T2 measurements in all areas. More specifically, the ICC values for the first rater ranged from 0.67 to 0.86, and for the second rater ranged from 0.64 to 0.82.

**Table 3:** Intraclass correlation coefficients (ICC) for the between measurements agreement, for each rater separately (intra-rater reliability) ** p ≤ 0.01; *** p ≤ 0.001 ICC: intraclass correlation coefficient; 95% CI: 95% confidence interval; SEM: standard error of measurement; MDC: minimal detectable change

	T1 vs T2 measurement			
	ICC (95% CI)	P	SEM	MDC
Rater 1				
Mastoid process (left)	0.81 (0.63-0.91)	<0.001^***^	0.85	2.35
Mastoid process (right)	0.67 (0.32-0.84)	0.001^***^	1.01	2.81
Bladder 10 (left)	0.70 (0.40-0.85)	<0.001^***^	1.05	2.92
Bladder 10 (right)	0.72 (0.43-0.86)	<0.001^***^	0.86	2.37
Zygapophyseal joint (left)	0.69 (0.36-0.84)	0.001^***^	1.18	3.27
Zygapophyseal joint (right)	0.72 (0.43-0.86)	<0.001^***^	0.97	2.70
Upper trapezius (left)	0.84 (0.67-0.92)	<0.001^***^	0.69	1.92
Upper trapezius (right)	0.78 (0.55-0.89)	<0.001^***^	0.87	2.42
Levator scapulae (left)	0.75 (0.48-0.87)	<0.001^***^	1.06	2.95
Levator scapulae (right)	0.75 (0.50-0.88)	<0.001^***^	1.10	3.05
Deltoid (left)	0.80 (0.60-0.90)	<0.001^***^	0.97	2.69
Deltoid (right)	0.83 (0.65-0.91)	<0.001^***^	0.94	2.61
Tibialis anterior (left)	0.80 (0.60-0.90)	<0.001^***^	1.06	2.95
Tibialis anterior (right)	0.86 (0.73-0.93)	<0.001^***^	0.90	2.48
Rater 2				
Mastoid process (left)	0.77 (0.54-0.89)	<0.001^***^	0.74	2.06
Mastoid process (right)	0.67 (0.33-0.84)	0.001^***^	0.78	2.16
Urinary bladder (left)	0.75 (0.49-0.88)	<0.001^***^	0.64	1.77
Urinary bladder (right)	0.74 (0.48-0.87)	<0.001^***^	0.66	1.84
Zygapophyseal joint (left)	0.70 (0.39-0.85)	<0.001^***^	0.86	2.38
Zygapophyseal joint (right)	0.67 (0.33-0.84)	0.001^***^	0.89	2.46
Upper trapezius (left)	0.64 (0.27-0.82)	0.003^**^	1.08	2.99
Upper trapezius (right)	0.82 (0.63-0.91)	<0.001^***^	0.69	1.90
Levator scapulae (left)	0.75 (0.50-0.88)	<0.001^***^	1.11	3.09
Levator scapulae (right)	0.74 (0.48-0.87)	<0.001^***^	0.92	2.56
Deltoid (left)	0.71 (0.42-0.86)	<0.001^***^	0.88	2.44
Deltoid (right)	0.77 (0.54-0.89)	<0.001^***^	0.74	2.05
Tibialis anterior (left)	0.74 (0.48-0.87)	<0.001^***^	1.06	2.95
Tibialis anterior (right)	0.72 (0.44-0.86)	<0.001^***^	1.00	2.78

ICC values for between raters’ agreement (inter-rater) are presented in Table 4. Significant agreement was found between the two raters at both time points Τ1 and T2. More specifically, the ICC values in T1 ranged from 0.68 to 0.92, and in T2 ranged from 0.68 to 0.89. 

**Table 4:** Intraclass correlation coefficients (ICC) for the between raters’ agreement, for each measurement separately (inter-rater reliability) *** p ≤ 0.001 ICC: intraclass correlation coefficient; 95% CI: 95% confidence interval; SEM: standard error of measurement; MDC: minimal detectable change

	T1				T2			
	ICC (95% CI)	P	SEM	MDC	ICC (95% CI)	P	SEM	MDC
Mastoid process (left)	0.92 (0.84-0.96)	<0.001^***^	0.49	1.35	0.89 (0.77-0.94)	<0.001^***^	0.60	1.66
Mastoid process (right)	0.92 (0.83-0.96)	<0.001^***^	0.43	1.20	0.84 (0.67-0.92)	<0.001^***^	0.65	1.79
Bladder 10 (left)	0.91 (0.82-0.96)	<0.001^***^	0.49	1.35	0.89 (0.77-0.94)	<0.001^***^	0.55	1.51
Bladder 10 (right)	0.88 (0.75-0.94)	<0.001^***^	0.51	1.42	0.84 (0.67-0.92)	<0.001^***^	0.58	1.62
Zygapophyseal joint (left)	0.91 (0.82-0.96)	<0.001^***^	0.56	1.56	0.81 (0.61-0.91)	<0.001^***^	0.81	2.24
Zygapophyseal joint (right)	0.87 (0.73-0.93)	<0.001^***^	0.60	1.68	0.74 (0.47-0.87)	<0.001^***^	0.88	2.43
Upper trapezius (left)	0.90 (0.80-0.95)	<0.001^***^	0.54	1.51	0.72 (0.44-0.86)	<0.001^***^	0.96	2.65
Upper trapezius (right)	0.78 (0.55-0.89)	<0.001^***^	0.80	2.21	0.75 (0.50-0.88)	<0.001^***^	0.89	2.47
Levator scapulae (left)	0.85 (0.69-0.92)	<0.001^***^	0.83	2.31	0.75 (0.50-0.88)	<0.001^***^	1.10	3.06
Levator scapulae (right)	0.75 (0.49-0.88)	<0.001^***^	1.02	2.82	0.68 (0.34-0.84)	0.001^***^	1.13	3.12
Deltoid (left)	0.77 (0.53-0.89)	<0.001^***^	0.88	2.45	0.76 (0.51-0.88)	<0.001^***^	0.98	2.71
Deltoid (right)	0.68 (0.34-0.84)	0.001^***^	1.11	3.08	0.73 (0.44-0.86)	<0.001^***^	1.00	2.78
Tibialis anterior (left)	0.85 (0.70-0.93)	<0.001^***^	0.86	2.39	0.77 (0.54-0.89)	<0.001^***^	1.08	2.98
Tibialis anterior (right)	0.75 (0.50-0.88)	<0.001^***^	1.09	3.02	0.68 (0.36-0.84)	0.001^***^	1.21	3.36

## Discussion

This study was designed to examine the intra- and inter-rater reliability of the Commander pressure algometer in patients with NSCNP.

The majority of the sample was women (66.7%), as women disproportionately report more neck/shoulder region musculoskeletal disorders than men [33].

Participants’ SFMPQ and depression (HADS) scores were significantly greater at T2, while the rest of the scale scores (VAS, PPI, GPE, and TSK) remained similar in T1 and T2. Since the questionnaires were repeated after six days, these findings may represent a variability in the intensity and quality of pain and depression-related symptoms the patients reported [12,13].

The findings showed “moderate to good” intra-rater reliability for both raters. ICC values for PPT at the seven bilaterally measured sites varied between 0.67 and 0.86 for the first rater (p ≤ 0.001) and 0.64 and 0.82 for the second rater (p ≤ 0.003). The inter-rater reliability was “moderate to excellent” with an ICC range from 0.68 to 0.92 in T1 and “moderate to good” with an ICC range of 0.68 to 0.89 in T2. The results of the present study adequately support the reliability, both intra- and inter-rater, of the Commander algometer at the selected measuring sites. Among the literature data, only one study has examined the reliability of the Commander algometer, which has found high intra-rater reliability (Cronbach’s alpha values range > 0.85) and moderate inter-rater reliability (ICC < 0.561) [9]. However, this study has applied Cronbach's a (which is a measure of internal consistency) as a test-retest reliability index.

In a broader comparison context on chronic neck patients, the ICC intra-rater values of this study are comparable to a number of studies [4,37,38]. Specifically, Persson et al. (2004) evaluated the test-retest reliability of PPT measurements in the upper trapezius muscle in 27 healthy women [37]. The range of the ICC was 0.7 to 0.9 which is close enough to our results. Oliveira et al. (2021) similarly found a “good intra-rater reliability” between measurements (ICC: 0.75-0.78) in women with chronic neck pain [38]. In Walton et al.'s (2011) study [4], the intra-rater reliability was higher than ours with the ICC values ranging from 0.94-0.97 to 0.96-0.97, both for healthy participants and patients with acute neck pain, respectively. Also, slightly higher intra-rater reliability was reported by two other studies on chronic neck pain patients [39,40]. In the first study, the ICC values ranged from 0.83 to 0.89 [39]. In the second study, the ICC ranged from 0.79 to 0.91 in the neck pain group [40].

The inter-rater reliability of PPT values in chronic neck pain patients of the present study is similar to findings gathered from related studies in the literature [4,38]. For instance, Walton et al. (2011) found “good reliability” in both healthy participants (ICC: 0.79-0.84) as well as participants with acute neck pain (ICC: 0.81-0.9) [4], while Oliveira et al. (2021), found a “good inter-rater reliability” (ICC: 0.858-0.874) on MTrPs of the upper trapezius muscle, in 30 young adult women with chronic neck pain [38]. The above findings confirm the reliability of the PPT measurements in chronic neck pain patients which is in line with the results of the current study, whose measurement method was the Commander algometer.

Among the study’s strengths was the assessment of central sensitization, achieved by measuring two distal or remote PPT sites (tibialis anterior muscle and the middle part of the deltoid muscle). A standardized methodology and a homogenous sample of chronic neck pain patients were also two strength factors. On the other hand, the subjective nature of the pain measurement can be viewed as a weakness in such studies [41]. The standardized procedure which ensured that patients would be allowed adequate time and participate in several trials to adapt and become acquainted and finally familiar with the procedures through which the measurements were taken can be considered an effective way to minimize the subjective aspect that could possibly affect the findings. Another interesting addition would be to include a sex and age-controlled group of healthy subjects to identify the differences between neck pain patients and healthy controls with the Commander algometer [4].

## Conclusions

The present study shows that the Commander algometer is a reliable tool for PPT measurements in Greek NSCNP patients. Both intra-rater and test-retest reliability were indicated for a period of six days in a Greek sample of patients suffering from NSCNP. The intra-rater reliability was "moderate to good” for both raters and the inter-rater reliability was “moderate to excellent” in T1 and “moderate to good” in T2. We conclude that pressure algometry using the Commander device is a suitable method for the assessment of PPT in NSCNP patients.
